# Microbial Diversity of a Mediterranean Soil and Its Changes after Biotransformed Dry Olive Residue Amendment

**DOI:** 10.1371/journal.pone.0103035

**Published:** 2014-07-24

**Authors:** José A. Siles, Caio T. C. C. Rachid, Inmaculada Sampedro, Inmaculada García-Romera, James M. Tiedje

**Affiliations:** 1 Department of Soil Microbiology and Symbiotic Systems, Estación Experimental del Zaidín, Consejo Superior de Investigaciones Científicas (CSIC), Granada, Spain; 2 Institute of Microbiology Paulo de Góes, Federal University of Rio de Janeiro, Rio de Janeiro, Brazil; 3 Center for Microbial Ecology, Michigan State University, East Lansing, Michigan, United States of America; 4 Thayer School of Engineering, Dartmouth College, Hanover, New Hampshire, United States of America; Virginia Tech, United States of America

## Abstract

The Mediterranean basin has been identified as a biodiversity hotspot, about whose soil microbial diversity little is known. Intensive land use and aggressive management practices are degrading the soil, with a consequent loss of fertility. The use of organic amendments such as dry olive residue (DOR), a waste produced by a two-phase olive-oil extraction system, has been proposed as an effective way to improve soil properties. However, before its application to soil, DOR needs a pre-treatment, such as by a ligninolytic fungal transformation, e.g. *Coriolopsis floccosa*. The present study aimed to describe the bacterial and fungal diversity in a Mediterranean soil and to assess the impact of raw DOR (DOR) and *C. floccosa*-transformed DOR (CORDOR) on function and phylogeny of soil microbial communities after 0, 30 and 60 days. Pyrosequencing of the 16S rRNA gene demonstrated that bacterial diversity was dominated by the phyla Proteobacteria, Acidobacteria, and Actinobacteria, while 28S-rRNA gene data revealed that Ascomycota and Basidiomycota accounted for the majority of phyla in the fungal community. A Biolog EcoPlate experiment showed that DOR and CORDOR amendments decreased functional diversity and altered microbial functional structures. These changes in soil functionality occurred in parallel with those in phylogenetic bacterial and fungal community structures. Some bacterial and fungal groups increased while others decreased depending on the relative abundance of beneficial and toxic substances incorporated with each amendment. In general, DOR was observed to be more disruptive than CORDOR.

## Introduction

The Mediterranean basin is one of the 25 most important biodiversity hotspots on Earth due to its particular climatic and geological characteristics [1]. This region has thus been identified as one of the priority regions for conservation in Europe, as human activity is causing a dramatic crisis in biodiversity [2]. However, as knowledge of soil microbial diversity in this part of the world is limited, it is essential to broaden our understanding of this diversity in order to achieve a balance between conservation and human development [3].

Olives are among the most important and widespread crops in the Mediterranean region, where they occupy a highly stable area of cultivation [4]. The olive oil industry generally produces vast amounts of wastes [5], and in Spain, the residues produced by the two-phase centrifuging olive oil extraction system has been highlighted. This technology produces a liquid phase (olive oil) and an organic waste sludge. Through a heating process and the use of organic solvents, this primary waste is then revalorized into low quality olive oil and a final waste product known as “alpeorujo” or dry olive residue (DOR) [6]. In Spain alone, 5 million tons of DOR are produced annually in a short period of time [7]. This waste, until now, has been used for energy and co-generation purposes [8]. However, the international regulations on limiting CO_2_ emissions and the presence of polyaromatic hydrocarbons in DOR combustion gases are restricting these practices [6], [8]. An alternative for DOR revalorization is its exploitation as an organic amendment as it contains high concentrations of organic matter and minerals of agricultural importance [9]. Its use as an organic amendment could be especially beneficial in the Mediterranean region, where many soils are experiencing degradation and fertility loss due to agro-chemical treatments, excessive and deep tillage, continuous cropping, overgrazing and luxury irrigation [10]. Organic amendments, which improve the physical, chemical and biological properties of soil, have thus been proposed as an effective way of maintaining and improving soil fertility [11]. However, DOR contains polyphenols and other organic components which are capable of inhibiting microbial growth, plant germination and morphogenesis [12], and therefore needs to be treated before being applied to soil in high doses. The transformation of DOR by ligninolytic fungi has been demonstrated to be a rapid and effective technique to stabilize organic matter, enhance C/N ratio, reduce phenolic concentration and to eliminate the phytotoxic effects of the waste in order to facilitate its use as an organic amendment [13]–[15].

Soil bacteria and fungi play a pivotal role in biogeochemical cycles and are responsible for nutrient cycling by mineralizing and decomposing of organic matter [3], [16], [17]. These communities may also influence nutrient availability for crops through solubilization, chelation and oxidation/reduction processes [18]. Furthermore, soil microorganisms establish symbiotic and antagonist relationships with plants that affect their status and perform other functions such as soil structure maintenance [19] and the degradation of pollutants [20]. Thus, microbial communities govern soil quality and constitute an important component of this ecosystem. In this sense, the implementation of sustainable soil strategies such as the use of biotransformed DOR as an organic amendment requires knowledge of microbial community behaviour under these conditions. To date, only Sampedro et al. [21] have made a preliminary study under “in vitro” conditions of the impact of *Phlebia* sp.-transformed DOR on soil microbiology using denaturing gradient gel electrophoresis (DGGE) and phospholipid fatty acid (PLFA) analysis. Other works have also assessed the effect of low doses of raw DOR on the physico-chemical properties of soil [9], . Thus, to the best of our knowledge, no studies have been made about the effect of raw and fungi-transformed DOR on soil microbiology, using more accurate and informative tools such as high-throughput sequencing techniques. In this survey, pyrosequencing was used to analyze the diversity of bacterial and fungal communities in a Mediterranean soil and their responses to raw and fungi-transformed DOR amendments. This work complements two other reports using the same experimental treatments but evaluated by culture-dependent approaches [23], [24]. In the present study, we aimed to: i) describe the bacterial and fungal diversity in an agricultural Mediterranean soil by means of 16S and 28S rRNA gene pyrosequencing, respectively; ii) obtain an insight into the functional changes produced by untransformed and *Coriolopsis floccosa*-transformed DOR on microbial communities over a short time period (0, 30 and 60 days) using a Biolog EcoPlate system; iii) and to investigate the effects of amendments containing these two types of DOR on soil fungal and bacterial communities.

## Materials and Methods

### Soil sampling

The soil studied, which was obtained from the “Cortijo Peinado” field (Granada, Spain, 37°13′N, 3° 45′W), was classified as loam according to the USDA system [25] and presented a low organic matter content (10 g kg^-1^ total organic carbon), which is typical of Mediterranean agricultural soils [4]. The main soil properties have been summarized in Table S1.

The climate in the region is typically Mediterranean with annual rainfall average of 357 mm, the wettest month is December with 53 mm and the driest one is August with 3 mm. The mean annual temperature is 15.1°C; the coldest month is January (mean 6.7°C) and warmest one is July (mean 24.8°C) (http://www.aemet.es).

The plot from which soil samples were collected has been used for agricultural purposes and, in recent years, fruit trees have been cultivated on this land. The area is not part of a conservation zone, does not contain any protected species and does not belong to a private land. Permission to sample the soil was obtained directly from the farm owners and technical experts. At sample collection time, the soil in the field had recently been ploughed and plants were not present. To collect the soil samples, the plot (10,000 m^2^) was divided into 10 sub-plots of equal size. Five 1 kg subsamples were collected randomly from the Ap horizon (at a depth of 0–20 cm) of each sub-plot and the subsamples were combined into a single pooled sample. Subsequently, the different composited samples were sieved (5 mm sterilized mesh) and mixed. The soil was stored for three days at room temperature until the experiment was performed.

### DOR

DOR was supplied by an olive oil manufacturer (Sierra Sur S.A., Granada, Spain) and was stored at −20°C until use.

### DOR biotransformation

DOR was transformed using the fungus *Coriolopsis floccosa* (Spanish Type Culture Collection, CECT 20449), formerly known as *Coriolopsis rigida*. The transformation was carried out according to Siles et al. [26]. Briefly, sterilized polyurethane sponge (PS) cubes were placed in Erlenmeyer flasks and 25 ml culture medium was added. *C. floccosa* inoculum was then added to the PS cubes and incubated at 28°C for 7 days. After this period of time, 25 g of DOR was placed above colonized PS. Solid-state cultures on DOR were carried out at 28°C for 30 days. Non-inoculated DOR samples were prepared as controls. Then, untransformed DOR (DOR) and *C. floccosa*-transformed DOR (CORDOR) were autoclaved three times for complete sterilization; sterility was confirmed by no observed growth on potato dextrose agar after 2 weeks. Finally, untransformed DOR (DOR) and *C. floccosa*-transformed DOR (CORDOR) were sieved, homogenized and stored at 4°C until the soil amendment experiment began. The main chemical properties of DOR and CORDOR have previously been reported by Siles et al. [27].

### Soil amendment

The experiment was carried out in 0.5 L pots. DOR and CORDOR were added to soil pots at concentrations of 50 g kg^-1^ (equivalent to 150 Mg ha^-1^). Soil samples without the residue were also prepared (control soil). One sorghum plant (*Sorghum bicolor*), of homogeneous size, was planted in each pot. The experiment was performed in a greenhouse with supplementary light at 25/19°C and 50% relative humidity. Manual regular watering was provided during the experiment, with soil humidity being maintained at 15–20%.

The untreated soil and soil amended with sterilized DOR and CORDOR were analysed at day 0, 30 and 60 of treatment. The experiment consisted of five pots for each treatment at each time. At each sampling, the soil from the five pots was mixed, homogenized and sieved (2 mm sterilized mesh). Three 100 g soil subsamples for each treatment were then placed in sterile Falcon tubes and stored at −80°C until the samples were analyzed.

### Community-level physiological profile

Community level physiological profiles (CLPP) were assessed using a Biolog EcoPlate system (BIOLOG. Inc., CA, USA). Each Biolog EcoPlate contains 31 different kinds of carbon sources in triplicate (seven types of carbohydrates, nine carboxylic acids, four polymers, six amino acids, two amines/amides and three miscellaneous types). To determine the CLPP for each sample, 1 g of soil was shaken in 10 ml of sterile saline solution (0.85% w/v NaCl) at 150 rpm for 1 h. Soil suspensions were then serially diluted on the basis of the viable cell counts obtained for each sample in Siles et al. [23], in order to avoid interference from the number of cells in the oxidation of substrates. 130 µl soil solutions were used for each well and Ecoplates were incubated at 25°C for 9 days. All analyses were performed in triplicate. The rate of use of C sources was indicated by the reduction in tetrazolium salts, which changed from colourless to purple [28]. Colour development for each well was obtained in terms of optical density (OD) at 590 nm every 24 h using an automated plate reader (Eon Microplate Spectrophotometer, BioTek Instruments, Inc., Germany). Microbial activity was then calculated as average well colour development (AWCD) as described by Insam et al. [29]. The 168 h OD data for each sample in triplicate, divided by their AWCD to normalize the values, were selected in order to determine substrate richness (*S_f_*), Shannon's functional diversity index (*H_f_*), substrate evenness (*J_f_*) and principal coordinate analysis (PCoA) using the PAST program ver. 2.17 [30]. PCoA of 9 samples according to their CLPP was carried out using normalized AWCD data for each substrate using a Euclidean distance matrix. Statistical differences between the treatments were analysed by ANOVA, and Tukey's honest significance difference (HSD) test was used for multiple comparison of means at a 95% confidence interval.

### DNA extraction, PCR amplification and pyrosequencing

Soil DNA was extracted using the MoBio Ultra Clean Soil DNA Isolation Kit (MoBio Laboratories Inc., Solana Beach, CA, USA) following the manufacturer's instructions. For each sample, three different DNA extractions were carried out, each of which was taken from a subsample. Afterwards, a pooled DNA sample for each treatment was prepared. All DNA templates were quantified with the aid of a Qubit 2.0 Fluorometer (Life Technologies, Grand Island, NY), and sample DNA concentrations were homogenised. Fungal and bacterial PCR amplifications were then carried out.

For bacteria, a 16S rRNA gene fragment was amplified capturing the hypervariable V4 region using the primers 577F and 926R designed with eight-base barcodes and pyrosequencing adapters [31]. Triplicate amplification reactions were performed in 20-µl volumes containing: 2 µl Roche 10 × Fast Start High Fidelity buffer with 18 mM MgCl_2_ (Roche Applied Sciences), 0.5 µl Roche Fast Start High Fidelity Taq (5 U/µl), 0.75 µl Invitrogen 10 mM deoxynucleoside triphosphate (dNTP) mix, 1 µl of each primer (10 pmol µl^−1^), 0.2 µl New England BioLabs 10 mg ml^−1^ bovine serum albumin (BSA), 3 µl DNA template (8 ng µl^−1^) and 11.55 µl H_2_O. Negative controls using sterilized water instead of soil DNA extract were included to check for primer and sample DNA contamination. The cycling conditions were as follows: initial denaturation at 94°C for 3 min, followed by 30 cycles of denaturation at 94 °C for 45 s, primer annealing at 56 °C for 45 s, extension at 72 °C for 1 min and final extension for 7 min. Reactions were then combined and purified using gel electrophoresis followed by the QIAquick gel extraction kit (QIAGEN Inc., Valencia, CA) and the QIAquick PCR Purification kit (QIAGEN Inc., Valencia, CA) according to the manufacturer's recommendations.

For fungi, a 625 bp fragment of the 28S rRNA gene was PCR-amplified in three replicate 20-µl reactions for each sample using primers LR3 and LR0 which included barcodes for sample discrimination [32]. PCR amplifications included: 4 µl Promega GoTaq buffer, 0.5 µl GoTaq DNA polymerase, 1.5 µl Roche 25 mM MgCl_2_, 1 µl Invitrogen 10 mM dNTP mix, 1 µl of each primer (10 pmol µl^−1^), 0.2 µl New England BioLabs 10 mg ml^−1^ BSA, 3 µl DNA template (8 ng µl^−1^) and 7.8 µl H_2_O. The thermal cycling program was: an initial denaturation at 94 °C for 3 min followed by 30 cycles at 94 °C for 1 min, at 51 °C for 40 s, and at 72 °C for 1 min, followed by an extension at 72 °C for 10 min. The reactions of each sample were then pooled and purifications were performed as for bacteria.

Amplicons from all samples for bacteria and fungi were composited together in equimolar concentrations and sequenced using a Roche Sequencer GS FLX Titanium series (454 Life Sciences, Branford, CT) at Utah State University.

### Pyrosequencing data analysis

Raw fungal and bacterial sequences were processed using Mothur version 1.31.0 [33]. Briefly, sequencing errors were reduced using the AmpliconNoise algorithm and low-quality sequences were removed [minimum length of 150 base pairs (bp), allowing 1 mismatch in the barcode, 2 mismatches in the primer, and homopolymers no longer than 8 bp]. Sequences were then aligned using the package's internal alignment feature and the SILVA database as template [34]. The chimera.uchime function was then used to identify potentially chimeric sequences which were subsequently removed [35]. Finally, the high-quality fungal and bacterial sequences were separately clustered into operational taxonomic units (OTUs) at a 3% dissimilarity distance. The number of sequences per sample was normalized before OTU definition based on the number of sequences obtained from the smallest library. OTU (phylogenetic richness – *S_p_*) distribution among samples was used to calculate rarefaction curves, the phylogenetic Shannon diversity index (*H_p_*), phylogenetic evenness (*J_p_*), Chao 1 and ACE (abundance-based coverage estimation) diversity estimator indices as well as Good's coverage by Mothur. Significant differences in Shannon diversity indices between control and amended samples at a given sampling time were assessed using the diversity *t* test, with p<0.05 being regarded as statistically significant [36]. On the other hand, differences in the fungal and bacterial community composition of each pair of samples were determined using the unweighted UniFrac metric (1,000 permutations). The unweighted UniFrac distances between samples were then used to model PCoA for each community. Finally, representative sequences from the 14 most abundant bacterial and fungal OTUs were obtained using Mothur. These sequences were identified by manually blasting in the EzTazon-e database (http://eztaxon-e.ezbiocloud.net/) [37] for bacteria and in the CBS-KNAW Fungal Biodiversity Center (http://www.cbs.knaw.nl/) for fungi.

To examine changes in the relative abundance of the different microbial groups mediated by amendments, the non-normalized bacterial and fungal sequences were classified using the Ribosomal Database Project (RDP) bacterial 16S rRNA gene and the fungal 28S rRNA gene classifier (http://rdp.cme.msu.edu/classifier/classifier.jsp) at a 50% bootstrap confidence level for both communities [32], [38], [39]. To describe the bacterial and fungal diversity of the soil studied, all 16S rRNA gene and 28S rRNA gene sequences were merged into a single file for each community, which was then subjected to the RDP classifier at the same bootstrap confidence level.

The raw pyrosequencing data were deposited in the MG-RAST public database (http://metagenomics.anl.gov/) under accession number 4552035.3 for bacteria sequences and 4552036.3 for fungi sequences.

Pearson's method was used to examine trends between functional and phylogenetic properties with respect to the chemical characteristics of the different soil samples reported in a previous article [27]. Normality of data was assessed by Kolmogorov–Smirnov test.

## Results

### Soil microbial diversity

#### Bacterial diversity

After pyrosequencing analysis, a total of 17,322 sequences across the 9 samples passed through the high quality filters with an average read length of 311 bp. The number of sequences per sample ranged from 2,248 (C-T0) to 1,674 (CORDOR-T1) (Table 1). The average number of bacterial sequences was 1,924±160 (mean±SD) per sample. These sequences were grouped into 2,267 different OTUs at 97% sequence similarity. This total number of OTUs consisted of 1,143 nonsingleton OTUs and 1,124 singletons. The rarefaction curves of the different treatments did not reach a plateau for any sample (Fig. S1). Good's coverage values (ranging from 0.76 to 0.81) (Table 1) also indicated that the sequences obtained were insufficient to fully capture bacterial diversity.

**Table 1:** Phylogenetic bacterial diversity characteristics obtained from unamended soil (C) and soil amended with untransformed DOR (DOR) or *C. floccosa*–transformed DOR (CORDOR) at 0 (T0), 30 (T1) and 60 (T2) days.

Soil sample	Sequence number	*S_p_*	*H_p_*	*J_p_*	Chao 1	ACE	Good's Coverage
C-T0	2248	649	5.82(5.76;5.89)	0.899(0.892;0.907)	1381(1202;1617)	2132(1946±2344)	0.76
DOR-T0	1957	580	5.59(5.52;5.66)	0.879(0.870;0.888)	1240(1070;1471)	1913(1739±2113)	0.79
CORDOR-T0	1981	592	5.59(5.52;5.66)	0.876(0.867;0.884)	1351(1156;1612)	2262(2059±2494)	0.78
C-T1	1858	613	5.70(5.63;5.77)	0.888(0.880;0.896)	1379(1185;1638)	2030(1845±2243)	0.77
DOR-T1	1904	522	5.34*(5.26;5.41)	0.853(0.843;0.863)	1103(945;1320)	1569(1416±1747)	0.81
CORDOR-T1	1674	646	5.81(5.74;5.88)	0.898(0.890;0.906)	1428(1235;1685)	2137(1949±2351)	0.76
C-T2	1787	560	5.84(5.77;5.90)	0.901(0.894;0.909)	1382(1203;1618)	2106(1922±2317)	0.76
DOR-T2	1888	638	5.81(5.74;5.87)	0.899(0.892;0.907)	1330(1160;1555)	2198(2006±2417)	0.77
CORDOR-T2	2025	651	5.86(5.80;5.93)	0.905(0.897;0.912)	1236(1094;1424)	1756(1602±1934)	0.77

Values in parenthesis are 95% confidence intervals as calculated using MOTHUR.

*S_p_* –Phylogenetic richness.

*H_p_*–Phylogenetic Shannon index.

*J_p_*–Phylogenetic evenness.

Diversity t test was performed for each amended sample with its control (* significant differences, p≤0.05).

**Figure 1:**
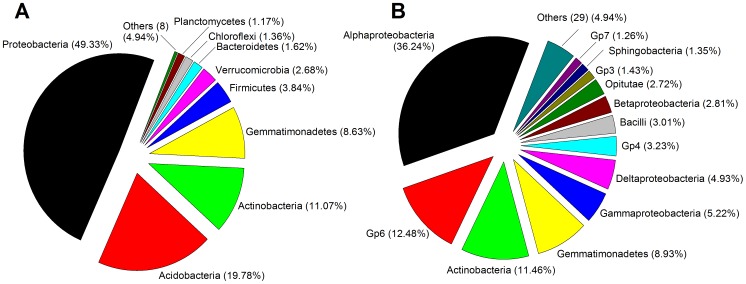
Composition of the bacterial community in the soil studied based on 16S rRNA gene pyrosequencing at phylum (A) and class (B) level.

Phylogenetic assignment analysis enabled the classification of ∼86% sequences at phylum level. The soil's bacterial diversity was distributed among 17 different phyla. The most common phyla were Proteobacteria, Acidobacteria, Actinobacteria, Gemmatimonadetes, Firmicutes and Verrucomicrobia (Fig. 1A). Approximately 83% of reads could be classified into 42 different classes, in particular, *Alphaproteobacteria* (with *Skermanella*, *Microvirga*, *Phenylobacterium* being the most common genera in this class), *Gp6* (*Gp6*), *Actinobacteria* (*Nocardioides* and *Solirubrobacter*), *Gemmatimonadete*s (*Gemmatimonas*), *Gammaproteobacteria* (*Steroidobacter*) and *Deltaproteobacteria* (*Geobacter*) (Fig. 1B).

#### Fungal diversity

A total of 38,410 valid 28S rRNA gene sequences, with an average read length of 338 bp, were obtained from the 9 samples. The average number of reads per sample was 4,267±2,258 (mean±SD), with sample C-T0 having the highest number of sequences (9,405) and DOR-T1 having the lowest number of reads (1,230) (Table 2). The total number of sequences represented a total of 1,160 different OTUs at 97% confidence threshold; 720 of these were nonsingleton OTUs and the rest (440) were singletons. The rarefaction curves (Fig. S2) and Good's coverage values (Table 2) indicated that sampling was not fully exhaustive for any sample. However, according to the coverage data, the fungal community was more thoroughly characterized than the bacterial community.

**Table 2:** Phylogenetic fungal diversity characteristics obtained from unamended soil (C) and soil amended with untransformed DOR (DOR) or *C. floccosa*–transformed DOR (CORDOR) at 0 (T0), 30 (T1) and 60 (T2) days.

Soil sample	Sequence number	*S_p_*	*H_p_*	*J_p_*	Chao 1	ACE	Good's Coverage
C-T0	9405	245	4.02(3.92;4.12)	0.731(0.726;0.757)	630(485;862)	994(854;1167)	0.87
DOR-T0	3658	240	3.91(3.80;4.01)	0.742(0.737;0.758)	625(479;890)	982(852;1092)	0.88
CORDOR-T0	2321	239	3.92(3.79;4.02)	0.700(0.698;0.728)	664(474;989)	918(784;1083)	0.89
C-T1	4093	247	4.16(4.07;4.26)	0.755(0.741;0.769)	557(443;737)	1055(917;1220)	0.88
DOR-T1	1230	235	4.01(3.91;4.11)	0.645(0.625;0.666)	586(455;792)	1272(1090;1494)	0.87
CORDOR-T1	4007	292	4.52*(4.61;4.77)	0.826(0.814;0.837)	610(499;782)	890(778;1027)	0.86
C-T2	4238	249	4.10(4.00;4.21)	0.744(0.728;0.760)	551(438;731)	838(724;979)	0.88
DOR-T2	4234	241	4.05(3.94;4.15)	0.738(0.722;0.755)	616(471;850)	1020(875;1197)	0.86
CORDOR-T2	5224	271	4.43*(4.34;4.52)	0.791(0.779;0.803)	735(565;1004)	1296(1128;1497)	0.86

Values in parenthesis are 95% confidence intervals as calculated using MOTHUR.

*S_p_* – Phylogenetic richness.

*H_p_*– Phylogenetic Shannon index.

*J_p_*– Phylogenetic evenness.

Diversity t test was performed for each amended sample with its control (* significant differences, p≤0.05).

**Figure 2:**
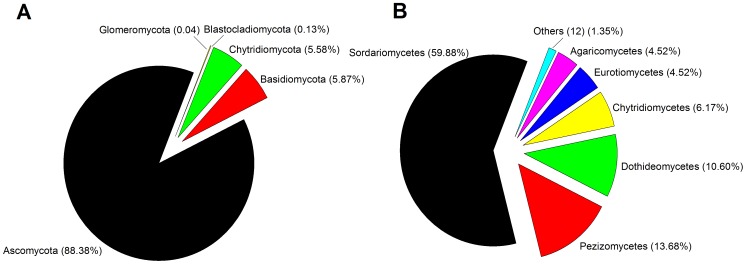
Composition of fungal community in the soil studied based on 28S rRNA gene pyrosequencing at phylum (A) and class (B) level.

Fungal RDP sequence classification (50% confidence threshold) yielded ∼80% classified sequences among 5 different phyla, particularly Ascomycota, Basidiomycota and Chytridiomycota (Fig. 2A). On the other hand, the fungal diversity of the fungal community consisted of 18 different classes (71% of total sequences). The most abundant classes were *Sordariomycetes* (with the most numerous genera in this class being *Chaetomium*, *Fusarium* and *Stachybotrys*), *Pezizomycetes* (*Ascobolus* and *Peziza*), *Dothideomycetes* (*Alternaria*, *Lophiostoma* and *Cladosporium*), *Chytridiomycetes* (*Nowakowskiella*), *Eurotiomycetes* (*Aspergillus* and *Eupenicillium*) and *Agaricomycetes* (*Coprinellus*) (Fig. 2B).

### Effects of DOR and CORDOR on soil microbial communities

#### Community level physiological profile (CLPP)

The functional indices *S_f_* and *H_f_* based on CLPPs significantly decreased (p<0.05) in the samples amended with DOR and CORDOR at 30 and 60 days (Table 3). On the other hand, *S_f_* and *H_f_* did not vary between samples at initial sampling time. The lowest microbial physiological diversity was found in the soil treated with CORDOR at 30 days (Table 3).

**Table 3:** Functional microbial diversity characteristics (mean±standard deviation) obtained from unamended soil (C) and soil amended with untransformed DOR (DOR) or *C. floccosa*–transformed DOR (CORDOR) at 0 (T0), 30 (T1) and 60 (T2) days.

Soil sample	*S_f_*	*H_f_*	*J_f_*
C-T0	27.33±0.58 cd	3.15±0.01 d	0.95±0.01 a
DOR-T0	22.50±3.54 bcd	3.00±0.11 d	0.96±0.01 a
CORDOR-T0	24.00±1.41 cd	3.05±0.05 de	0.96±0.01 a
C-T1	24.00±1.41 de	3.00±0.01 e	0.95±0.01 a
DOR-T1	19.50±0.71 ab	2.84±0.01 c	0.94±0.01 a
CORDOR-T1	17.50±2.12 a	2.49±2.49 a	0.92±0.03 a
C-T2	24.50±0.71 de	3.12±0.02 e	0.97±0.01 a
DOR-T2	20.50±0.71 abc	2.79±0.02 c	0.94±0.01 a
CORDOR-T2	17.00±1.41 a	2.64±0.07 b	0.94±0.02 a

*S_f_* –Functional richness.

*H_f_*–Functional Shannon index.

*J_f_*–Functional evenness.

For each variable, data followed by different letter are significantly different according to Tukey's HSD test (P≤0.05).

**Figure 3:**
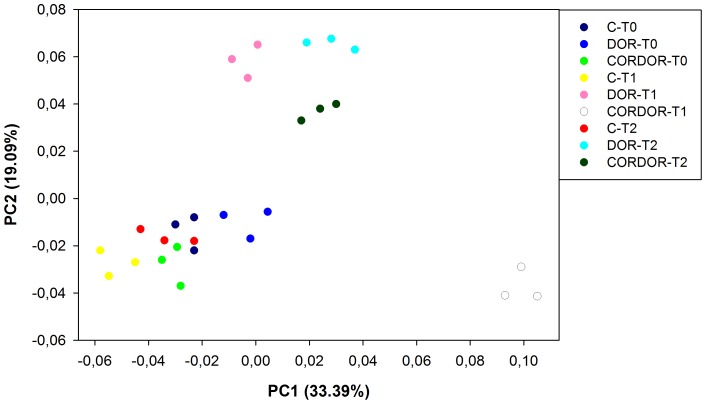
PCoA based on Euclidean distances of community level physiological profile (CLPP) dataset for unamended soil (C) and soil amended with untransformed DOR (DOR) or *C. floccosa*–transformed DOR (CORDOR) at 0 (T0), 30 (T1) and 60 (T2) days. Percent variability explained by each principal coordinate is shown in parentheses after each axis legend.

PCoA of the CLPP dataset showed that around 53% of the variability was due to two principal coordinates, the first (PC1) accounting for 33.39% and the second (PC2) accounting for 19.09% (Fig. 3). These two coordinates grouped the samples in two clusters and one sample was situated alone. The values for correlating each C source with PC1 and PC2 are shown in the Table S2. One of the clusters, situated in the lower-left quadrant, contained all the samples at initial sampling time and control samples at 30 and 60 days, with this group being closely associated with carbohydrates and polymers (D-cellobiose, cyclodextrin and glycogen). Another cluster, located in the upper quadrants, consisted of the samples amended with DOR for 30 and 60 days and soil amended with CORDOR for 60 days. This group was highly weighted by carboxylic acids (malic, itaconic and D-galacturonic acid) and carbohydrates (Beta-methyl-D-glucoside). Finally, the soil treated with CORDOR for 30 days was situated in the lower-right quadrant; some carbohydrates (D-xylose and i-erythritol), amines/amides (putrescine and phenylethylamine) and amino acids (L-arginine) were the most oxidized substrates in this sample.

#### Phylogenetic bacterial community

The DOR amendment significantly reduced *H_p_* (diversity *t* test, p<0.001) at 30 days with respect to the unamended soil (Table 1). This amendment also caused a diminution in the *S_p_*, *J_p_*, Chao 1 and ACE indices at this sampling time. However, DOR did not alter bacterial diversity at the other sampling times. In addition, no changes were observed in the diversity characteristics of samples amended with CORDOR at any sampling time (Table 1).

**Figure 4:**
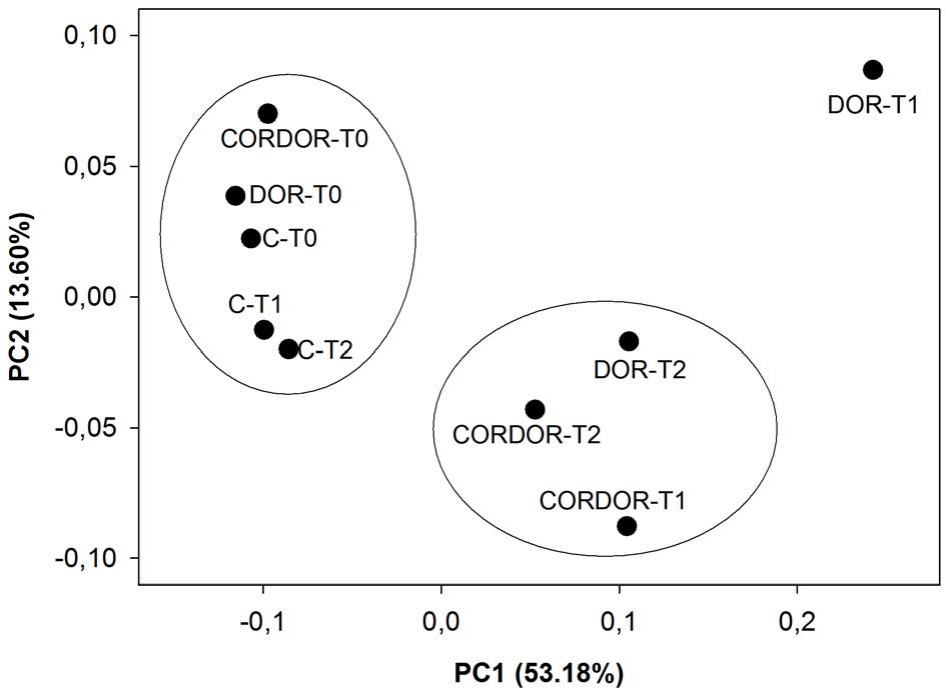
PCoA based on unweighted UniFrac distances of bacterial community found in unamended soil (C) and soil amended with untransformed DOR (DOR) or *C. floccosa*–transformed DOR (CORDOR) at 0 (T0), 30 (T1) and 60 (T2) days. Percent variability explained by each principal coordinate is shown in parentheses after each axis legend.

PCoA of bacterial pyrosequencing data based on the unweighted UniFrac metric revealed that amendments caused variations in community structure (Fig. 4). The analysis showed that 66.78% of variance can be explained by two principal coordinates, one accounting for 53.18% and the other for 13.60% of the variation. The nine samples grouped in two clusters and one sample did not cluster. One of the groups was situated to the left of PC1 and consisted of all samples at initial sampling time and control samples at 30 and 60 days. The pairwise unweighted UniFrac test did not find any significant differences between the samples of this group (p>0.05). Another group was situated in the lower right quadrant and was made up of the samples amended with CORDOR for 30 and 60 days as well as the soil treated with DOR for 60 days. The bacterial community structure in these samples was significantly different with respect to their control samples (pairwise unweighted UniFrac test, p<0.001). Finally, soil amended with DOR for 30 days was located in the upper right quadrant. This sample showed significant differences with respect to unamended soil at 30 days (p<0.001).

**Figure 5:**
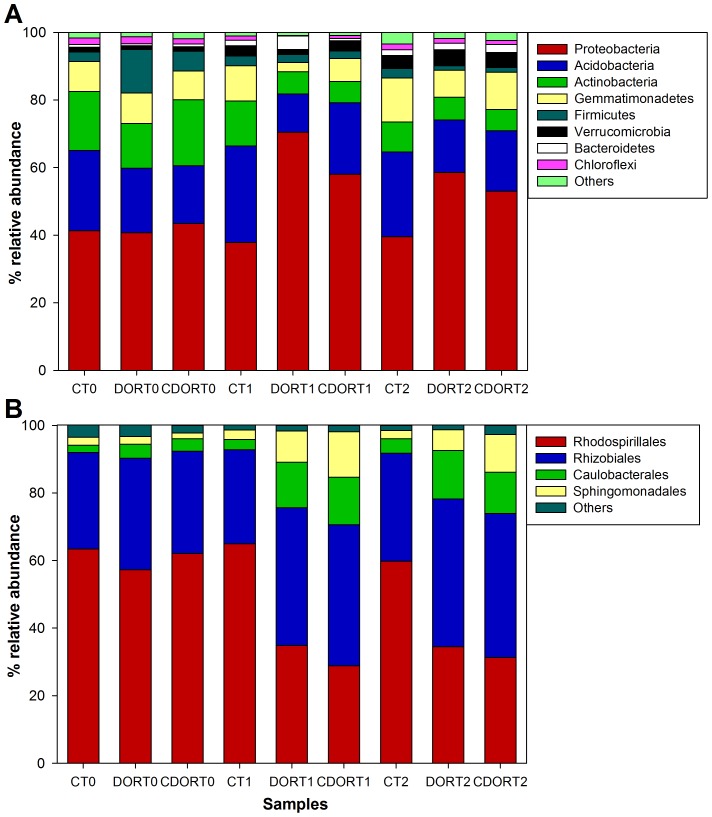
Relative abundance of the different bacterial phyla (A) and orders of *Alphaproteobacteria* (B) found in unamended soil (C) and soil amended with untransformed DOR (DOR) or *C. floccosa*–transformed DOR (CDOR) at 0 (T0), 30 (T1) and 60 (T2) days.

Changes in bacterial community structures mediated by amendments were probably caused by alterations in the relative abundance of the Proteobacteria, Acidobacteria, Actinobacteria and Gemmatimonadetes phyla (Fig. 5A). In the Proteobacteria phylum, the most significant changes occurred in *Alphaproteobacteria* due to its predominance in this phylum (Fig. S3A). It is interesting to note that the orders belonging to *Alphaproteobacteria* responded differently to amendments (Fig. 5B), since *Rhodospirillales* [represented in the top 14 most abundant bacteria with OTU 1 (*Skermanella stibiiresistens*) and OTU 9 (*Skermanella aerolata*) (Table S3)] decreased their relative abundances after DOR and CORDOR application at 30 and 60 days while *Rhizobiales* [OTU 2 (*Microvirga aerophila*) and OTU 7 (*Rhizobium rosettiformans*) (Table S3)], *Caulobacterales* [OTU 6 (*Phenylobacterium* sp.) (Table S3)] and *Sphingomonadales* showed considerably greater abundance after these treatments at the same sampling times. With regard to the Acidobacteria phylum, an overall decrease in its abundance in amended samples was observed, with the reduction being more evident in samples amended with DOR (Fig. 5A). *Gp6* and *Gp7* were the classes most affected by inputs although clear evidences of the specific effect of each amendment on these groups were not found (Fig. S3B). Four of the most abundant OTUs (3, 8, 10 and 12) were identified as uncultured *Acidobacteria* although a more detailed identification of these OTUs was not possible (Table S3). In the *Actinobacteria* phylum, the suborder *Propionibacterinae* [OTU 5 (*Nocardoides mesophilus*) (Table S3)] responded negatively to both types of amendment at 30 and 60 days (Fig. S3C). Finally, Gemmatimonadetes were also affected by the application of DOR. OTU 13, associated with *Gemmatimonadaceae,* the only family present in this phylum, experienced a drastic decrease in its relative abundance after treatment with DOR at 30 and 60 days (Table S3).

#### Phylogenetic fungal community

With regard to fungal community, CORDOR caused a significant increment in *H_p_* as compared to unamended samples (diversity t test, p<0.05) after 30 and 60 days (Table 2). The other diversity indices were also affected by this amendment. By contrast, DOR did not alter the diversity characteristics of fungal community at any sampling time.

**Figure 6:**
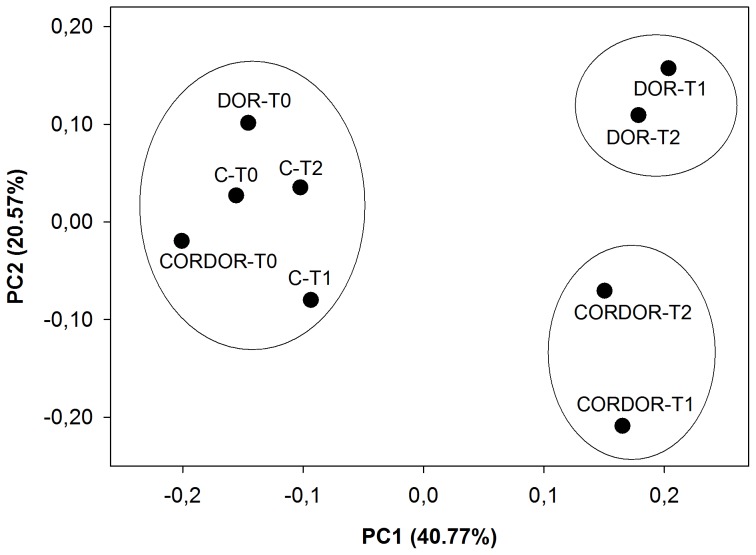
PCoA based on unweighted UniFrac distances of fungal community found in unamended soil (C) and soil amended with untransformed DOR (DOR) or *C. floccosa*–transformed DOR (CORDOR) at 0 (T0), 30 (T1) and 60 (T2) days. Percent variability explained by each principal coordinate is shown in parentheses after each axis legend.

Fungal PCoA based on unweighted UniFrac distances indicated that this community structure was altered depending on the amendment applied (Fig. 6). The two principal PCoA coordinates explained 61.34% of the variations (40.77% and 20.57%, respectively) and separated the 9 samples into three groups; one group, in the left quadrant, was made up of all the samples at initial sampling time and control samples at 30 and 60 days. No significant differences (p>0.05) between these samples were found using the pairwise unweighted UniFrac test. Another group, in the upper right quadrant, consisted of DOR amended samples at 30 and 60 days; the fungal community structure of these samples differed significantly from their respective control samples (pairwise unweighted UniFrac test, p<0.001). The last group, in the lower right quadrant, included the samples amended with CORDOR for 30 and 60 days, which also presented a significantly different fungal community structure from that of the respective unamended samples (pairwise unweighted UniFrac test, p<0.001).

**Figure 7:**
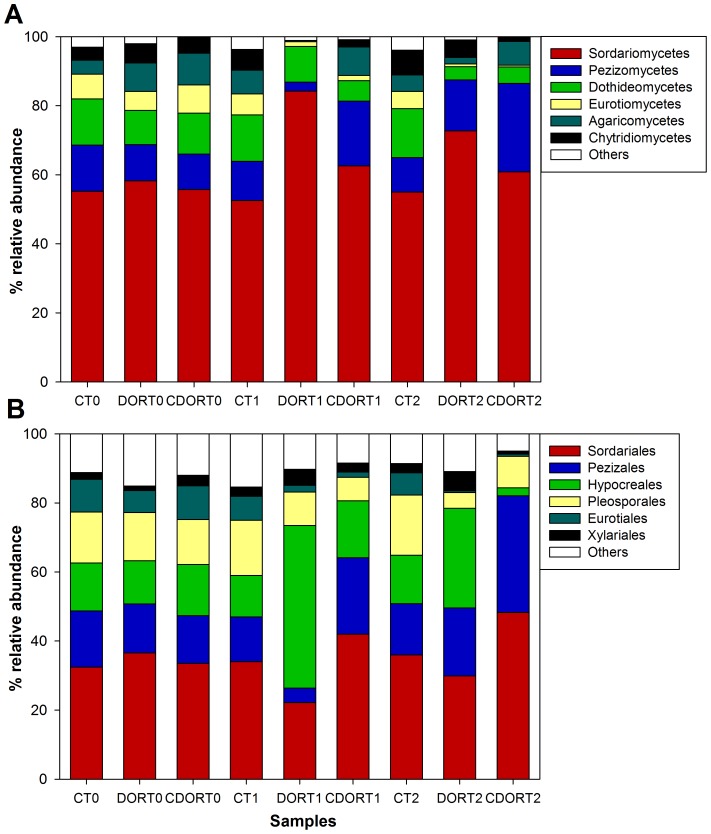
Relative abundance of the different fungal classes (A) and orders (B) found in unamended soil (C) and soil amended with untransformed DOR (DOR) or *C. floccosa*–transformed DOR (CDOR) at 0 (T0), 30 (T1) and 60 (T2) days.

The most striking changes in this community caused by soil amendments occurred in the Ascomycota phylum which dominated fungal diversity (Fig. S4). In this sense, the *Sordariomycetes* class responded positively to both types of amendment after 30 and 60 days, with a more important increase being observed following treatment with DOR (Fig. 7A). This increment in the samples amended with DOR for 30 and 60 days was due to an increase in the relative abundance of *Hypocreales* (Fig. 7B), which was probably caused by OTU 1 (*Fusarium* sp.) (Table S4). Curiously, this group decreased following CORDOR amended treatment for 30 and 60 days (Fig. 7B). However, in these treatments, *Sordariales* increased, probably due to a proliferation of genera such as *Podospora* sp. (OTU 11) and *Cercophora* sp. (OTU12), although, in this group, CORDOR caused a reduction in *Chaetomium* sp. (OTU 2) (Table S4). DOR also caused a reduction in this fungal group after 30 and 60 days. On the other hand, the *Eurotiales* order (*Eurotiomycetes* class) also decreased following the application of both types of amendment after 60 days (Fig. 7B). These changes could be attributed to a diminution in *Aspergillus terreus* (OTU 8), among others (Table S4). In the *Dothideomycetes* class, there was a particularly sharp decrease in *Pleosporales* [OTU 3 (*Preussia terricola*)] in the amended samples after 30 and 60 days. With regard to *Pezizomycetes* (*Pezizales* order), OTU 14 (*Ascobolus*), increased following treatment with CORDOR, especially after 60 days (Table S4). Finally, it is interesting to note that OTU 9, identified as *Cryptococcus* sp. (Basidiomycota), and OTU 13, identified as *Coprotus ochraceus* (*Leotiomycetes*, Ascomycota), also responded positively to DOR and CORDOR treatments after 30 and 60 days (Table S4).

## Discussion

### Soil microbial diversity

Human actions are causing a biodiversity crisis, with species extinction rates up to 1000 times higher than background rates [2]. Conservation strategies are therefore necessary, especially in vulnerable zones such as the Mediterranean biome, which is currently considered to be one of the most vulnerable of the Earth's thirteen terrestrial biomes [3]. In this context, acquiring knowledge of soil biodiversity would be a first step in the development of sustainable activities.

Our results demonstrated that microbial communities in this type of soil are extremely diverse, with high values for richness estimators recorded in each sample. This was corroborated by the high proportion of unclassified sequences at phylum level despite the use of a 50% threshold for read classification [40]. The phylum composition of bacterial communities in this soil was consistent with previous pyrosequencing surveys of soils in the Iberian Peninsula [41]–[43]. Although our study found a higher proportion of Acidobacteria, especially in the Gp6 class, than the aforementioned works, this could be related to the high alkaline pH (∼8.4) of soil [44]. Higher relative abundance levels were found for *Gemmatimonadetes* (8.63%) than the normal levels reported in other soils [45], which could be due to the xeric conditions of Mediterranean basin soils [46]. On the other hand, it is not surprising that our results for bacterial diversity differed substantially from data obtained for the present experiment using culture-dependent techniques. To carry out the culture-dependent study, 900 strains were isolated, clustered by their fatty acid methyl ester profiles and groups of isolates were identified by partial sequencing of 16S rRNA gene [23]. Culturable bacterial diversity was distributed between Actinobacteria (50.6%), Proteobacteria (40.4%), Firmicutes (4.5%) and Bacteroidetes (4.4%). Other studies have also shown that important differences in the bacterial diversity of an environment are observed when culture–dependent and culture–independent studies are carried out simultaneously [47]–[49]. These discrepancies are understandable given the difficulty in obtaining culturable members of certain bacterial groups. In this respect, it is interesting to note that, despite the high abundance levels of Acidobacteria in this soil and the proven effectiveness of VL 70 medium with regard to Acidobacteria isolation [50], it was not possible to obtain any strain belonging to this phylum. The absence of culturable Acidobacteria in this soil may be due to the slow growth of these bacteria or the inhibition in the development of these colonies caused by other culturable bacteria [47]. It is also worth noting that Actinobacteria was the most common culturable phylum, while pyrosequencing data showed that Proteobacteria dominated bacterial diversity in this soil. This bias may be a consequence of the copiotrophic lifestyle of some Actinobacteria groups [51]. Pyrosequencing data showed that genera such as *Microvirga*, *Nocardioides* and *Rhizobium* were among the most abundant in this Mediterranean soil, and it is interesting to note that numerous isolates belonging to these genera were found in the previous culture-dependent survey [23]. In this way, VanInsberghe et al. [49], in a work assessing bacterial diversity using culture-dependent and pyrosequencing techniques, suggested that the combination of both approaches may be useful as the isolates obtained can be used for genomic and physiological research.

Ascomycota clearly dominated the fungal diversity of the soil studied, a finding which is in line with other pyrosequencing studies of Mediterranean soils [3], [42], [52]. This research also demonstrated that Basidiomycota and Chytridiomycota, though in lower concentrations than Ascomycota, may also be found in these environments. The limited presence of Glomeromycota (phylum associated with arbuscular mycorrhiza) in the present work is worth noting, which may be due to the absence of plants in the plots when soil samples were collected. The fungal pyrosequencing-based diversity found in the present research was consistent with the diversity obtained by the application of culturable-dependent techniques to these soil samples. In this culture-dependent survey, 1,733 strains were obtained and characterized with the aid of morphological and molecular techniques [24], with the majority of isolates being distributed among the *Sordariomycetes*, *Eurotiomycetes* and *Dothideomycetes* classes [24]. It is interesting to note that some of the most abundant fungal species (*Chaetomium, Fusarium, Aspergillus*, *Alternatia* and *Cladosporium*) in pyrosequencing data were also the most common culturable fungi [24]. These findings are not surprising as soil fungi are dominated by readily culturable saprobic filamentous forms [53]. Similarly, Klaubauf et al. [54] have reported that data from both molecular-based and culture-dependent techniques correlate more closely in soil fungal communities than in bacterial communities.

### Effects of DOR and CORDOR on soil microbial communities

The present survey assessed the impact of DOR and CORDOR as organic amendments on the soil microbial community. However, the application of raw DOR is not possible as it causes oxidative stress in plants and presents considerable phytotoxic activity due to its high phenol content and other substances such as fatty acids [55]. In our greenhouse experiment, the phytotoxicity of DOR at agronomic rates (150 Mg ha^−1^) was confirmed; DOR produced a drastic reduction in the shoot dry weight of sorghum plants, while CORDOR reduced toxicity levels [23]. These results were also corroborated by a sorghum field-based experiment involving both amendments (unpublished data). Other studies have reported that the application of raw DOR to olive groves did not lead to a diminution in olive production over the long term, although only small doses of DOR were applied in this experiment (27 and 54 Mg ha^−1^) [6]. However, other works have shown that olive oil wastewater (OMW), a liquid residue obtained from a three-phase olive-mill extraction system with a composition similar to that of DOR and produced in other Mediterranean countries such as Italy and Greece, can cause olive grove death (800 m^3^ ha^−1^) and negatively affect the quality of olive oil at high doses [56], [57]. Thus, the phytotoxic activity of DOR depends on the doses applied, with seasonal crops being especially susceptible to these effects.

Previous studies have assessed the impact of raw and transformed DOR as well as OMW on soil microbiology using DGGE, PLFA and colony forming unit (CFU) counts [21], [58]–[61]. Thus, our understanding of the changes produced by DOR and OMW (olive mill waste) in the phylogenetic composition of microbial communities is limited, although these studies have demonstrated that OMW has a beneficial effect on microbial abundance. Previous surveys have found, through the use of culture-independent techniques, that DOR caused a rapid and marked increase in bacterial and fungal abundance in the soil analyzed in the present work after 30 and 60 days of treatment, while CORDOR resulted in slower and more moderate increases [23], [26]. Thus, although microtoxic effects have been associated with raw olive mill waste [62], [63], it was not possible to detect these effects in relation to abundance levels in an environment as diverse and complex as soil. The beneficial impact of this waste on certain microbial groups, due to the input of easily degradable compounds, probably masked potential microtoxicity. In this respect, previous studies have established that the impact of olive wastes on soil microbiology is the result of complex, sometimes contradictory, effects, and depends on the relative amounts of beneficial and toxic organic and inorganic compounds added [57], [64].

CLPP analyses showed a diminution in functional diversity (*S_f_* and *H_f_*) and changes in the functional structure of microbial communities after amendments application, especially in the case of CORDOR after 30 days. Some studies, using the Biolog system, have shown an increase or no change in functional diversity after the application of amendments [65], while others have demonstrated a reduction in this diversity [42]. These differences between works are probably due to variations in the kinds of organic amendments used. In the present study, the high level of functional diversity in the soil studied can be regarded as normal in relation to agronomical soil [66]. The subsequent diminution in diversity following the application of amendments could be due to the adaptation and selective proliferation of certain microorganisms at the expense of the incorporated nutrients since functional diversity negatively correlated with total organic C (R_pearson_ -0.814, P<0.05) and total N (R_pearson_ -0.729, P<0.05). The different functional community structure found in the soil amended with CORDOR after 30 days as compared to the other amended samples may be a consequence of the different nutrients added, as this type of treatment was especially characterised by the oxidation of C sources containing N (amino acids and amines/amides). The ability of some saprobic fungi to increase N content in DOR during bioremediation has been reported in previous studies [14].

However, no dramatic changes were caused by DOR and CORDOR in phylogenetic microbial diversity with respect to the unamended samples, with DOR reducing bacterial diversity after 30 days and CORDOR increasing fungal diversity after 30 and 60 days. Thus, it is possible to conclude that the most significant effects of the amendments on soil microbiology are related to alterations in the community structure. Similar conclusions were reached in the culture-dependent studies of this experiment. [23], [24]. In the case of the bacterial community, the most important change mediated by amendments was an increase in Proteobacteria, which was more marked in the case of DOR at 30 days. A detailed analysis of this phylum found that the most significant changes occurred in the *Alphaproteobacteria* class. *Rhodospirillales* decreased with the addition of DOR and CORDOR after 30 and 60 days. In fact, a negative correlation between organic C and the relative abundance of *Rhodospirillales* (R_pearson_ -0.953, P<0.001) was found, probably due to the adaptation of this bacterial group to oligotrophic nutritional conditions [67]. On the other hand, *Rhizobiales*, *Caulobacterales* and *Sphingomonadales* increased with the addition of DOR and CORDOR after 30 and 60 days due to their saprophytic lifestyle and their capacity to degrade recalcitrant compounds, even phenols [68], [69]. There was a positive correlation between the relative abundance of these groups and total soil organic C (R_pearson_ 0.945, P<0.001). It is remarkable the increase of *Rhizobiales* with both amendments, as this group contains species associated with nitrogen fixation and plant growth promotion [70], which may be a beneficial aspect of the application of these amendments to soil. By contrast, given that Acidobacteria have been identified as oligotrophic bacteria [71] and alkaline soil inhabitants, especially subgroups 5, 6 and 7 [44], the increase in nutrients and decrease in pH observed in soil after applications of DOR and CORDOR could explain the diminution in the relative abundance of this group. Unlike the aforementioned bacterial groups, there are previous works assessing the impact of olive mill wastes on Actinobacteria, which reported a positive effect of OMW on this bacterial group over the short term [58], [72]. However, with the aid of DGGE, Karpouzas et al. [73] suggested that OMW is responsible for dramatic alterations in this bacterial community. Siles et al. [23] found that culturable Actinobacteria responded differently to DOR and CORDOR depending on the phylogenetic group considered. In the present study, the changes in this community mediated by the amendments were limited with the exception of members of the *Propionibacterinae* suborder, which decreased with DOR and CORDOR after 30 and 60 days. Using a culturable-dependent approach, Siles et al. [23] also found that *Streptomyces* spp. were negatively affected by DOR and that the application of CORDOR offset this adverse effect. In the present survey, it was not possible to obtain conclusive results concerning this bacterial group as the number of sequences obtained belonging to *Streptomyces* sp. was very low. In this respect, these findings are in line with the research carried out by Shade et al. [48], where culture dependent studies were regarded as useful for assessing the soil rare biosphere.

For the fungal community, it is worth noting the findings obtained with respect to *Fusarium* spp., whose relative abundance increased with the addition of DOR. Siles et al. [24] have also reported an increase in culturable *Fusarium* spp. after the application of DOR, which is a reasonable finding given that *Fusarium* spp. have been associated with lignocellulosic wastes due to their saprophytic lifestyle [74]. This may be a drawback for the application of this residue in its raw state, which may adversely affect crop development as some species of *Fusarium* have been identified as potential phytopathogens [75]. On the other hand, CORDOR treatments led to a decrease in this fungal group. Previous studies have also demonstrated the suppressive effect of composted OMW on certain fungal phytopathogen species [76]. *Aspergillus terreus* and *Cryptoccocus* sp. were also observed to decrease and increase, respectively, following the application of amendments, which is in line with the culture-dependent study [24]. Curiously, Karpouzas et al. [59] observed an increment in *Cryptococcus* sp. after OMW application to soil, which was explained as the result of the ability of these microorganisms to metabolize a high variety of substrates. On the other hand, we observed a striking reduction in the relative abundance of *Chaetomium* spp., which are saprobic fungi and potential degraders of cellulosic material, with both amendments after 30 and 60 days [77]. This could be explained by the high sensitivity of this group's members to phenols [78]. In this respect, a negative correlation was found between soil phenol content and the number of *Chaetomium* sp. sequences (R_pearson_ -0.759, P<0.05). However, CORDOR presented a lower phenol content than DOR due to its transformation by *C. floccosa* [27]. Thus, the decrease in *Chaetomium* sp., in amended samples, could also be explained by the ability of other microbial groups positively affected by inputs to inhibit their proliferation [79]. By contrast, other fungal groups such as *Podospora* sp. and *Cercophora* sp., which have been identified as coprophilous fungi [80] and have been associated with lignocellulosic material [81], [82], were found to increase after 30 and 60 days following the addition of CORDOR, thus supporting the hypothesis that olive mill wastes affects soil microbiology in contradictory ways.

In conclusion, this work has showed that the Mediterranean soil analyzed has an incredible microbial diversity. The application of DOR and CORDOR resulted in a diminution in functional diversity as well as changes in functional community structures depending on the kind of treatment applied given the different types of C sources provided. In addition to its phytotoxicity, DOR was shown to be more disruptive than CORDOR in relation to bacterial and fungal communities as the impact of olive mill wastes on soil microbial communities depends on the relative amounts of beneficial and inhibitory components added, which alter nutrient and toxic substance levels and chemical soil properties. Although a direct link cannot be established, the bacterial (*Rhizobiales*, *Caulobacterales* and *Sphingomonadales*) and fungal (*Fusarium* sp., *Cryptococcus* sp., *Podospora* sp. and *Cercophora* sp.) groups that benefited most from amendments, are probably responsible for changes in soil functionality.

## Supporting Information

Figure S1 Bacterial rarefaction curves. Rarefaction curves for bacteria obtained from unamended soil (C) and soil amended with untransformed DOR (DOR) or *C. floccosa*–transformed DOR (CORDOR) at 0 (T0), 30 (T1) and 60 (T2) days.
(TIF)

Figure S2 Fungal rarefaction curves. Rarefaction curves for fungi obtained from unamended soil (C) and soil amended with untransformed DOR (DOR) or *C. floccosa*–transformed DOR (CORDOR) at 0 (T0), 30 (T1) and 60 (T2) days.
(TIF)

Figure S3 Changes in bacterial community mediated by amendments. Relative abundance of the different *Proteobacteria* classes (A), *Acidobacteria* classes (B) and *Actinobacteria* suborders (C) found in unamended soil (C) and soil amended with untransformed DOR (DOR) or *C. floccosa*–transformed DOR (CDOR) at 0 (T0), 30 (T1) and 60 (T2) days.
(TIF)

Figure S4 Changes in fungal community mediated by amendments. Relative abundance of the different fungal phyla found in unamended soil (C) and soil amended with untransformed DOR (DOR) or *C. floccosa*–transformed DOR (CDOR) at 0 (T0), 30 (T1) and 60 (T2) days.
(TIF)

Table S1 Soil chemical properties. The chemical properties of the soil used in the study (mean±standard deviation).
(DOCX)

Table S2 Correlation of carbon sources with principal coordinates. Correlation of carbon sources with the first (PC1) and second principal coordinates (PC2) after principal coordinate analysis (PCoA) of community level physiological profiles (CLPP) from unamended soil and soil amended with untransformed DOR or *C. floccosa*–transformed DOR at 0, 30 and 60 days.
(DOCX)

Table S3 Identification and abundance of the major bacterial OTUs. Basic information of the bacterial 14 most abundant OTUs and their relative abundance (percent) in unamended soil (C) and soil amended with untransformed DOR (DOR) or *C. floccosa*–transformed DOR (CORDOR) at 0 (T0), 30 (T1) and 60 (T2) days.
(DOCX)

Table S4 Identification and abundance of the major fungal OTUs. Basic information of the fungal 14 most abundant OTUs and their relative abundance (percent) in unamended soil (C) and soil amended with untransformed DOR (DOR) or *C. floccosa*–transformed DOR (CORDOR) at 0 (T0), 30 (T1) and 60 (T2) days.
(DOCX)
