# Supplementary figures and images for: Microbial Diversity of a Mediterranean Soil and Its Changes after Biotransformed Dry Olive Residue Amendment
Source: PLoS One. 2014 Jul 24;9(7):e103035. doi: 10.1371/journal.pone.0103035 (PMC4109964; doi:10.1371/journal.pone.0103035)

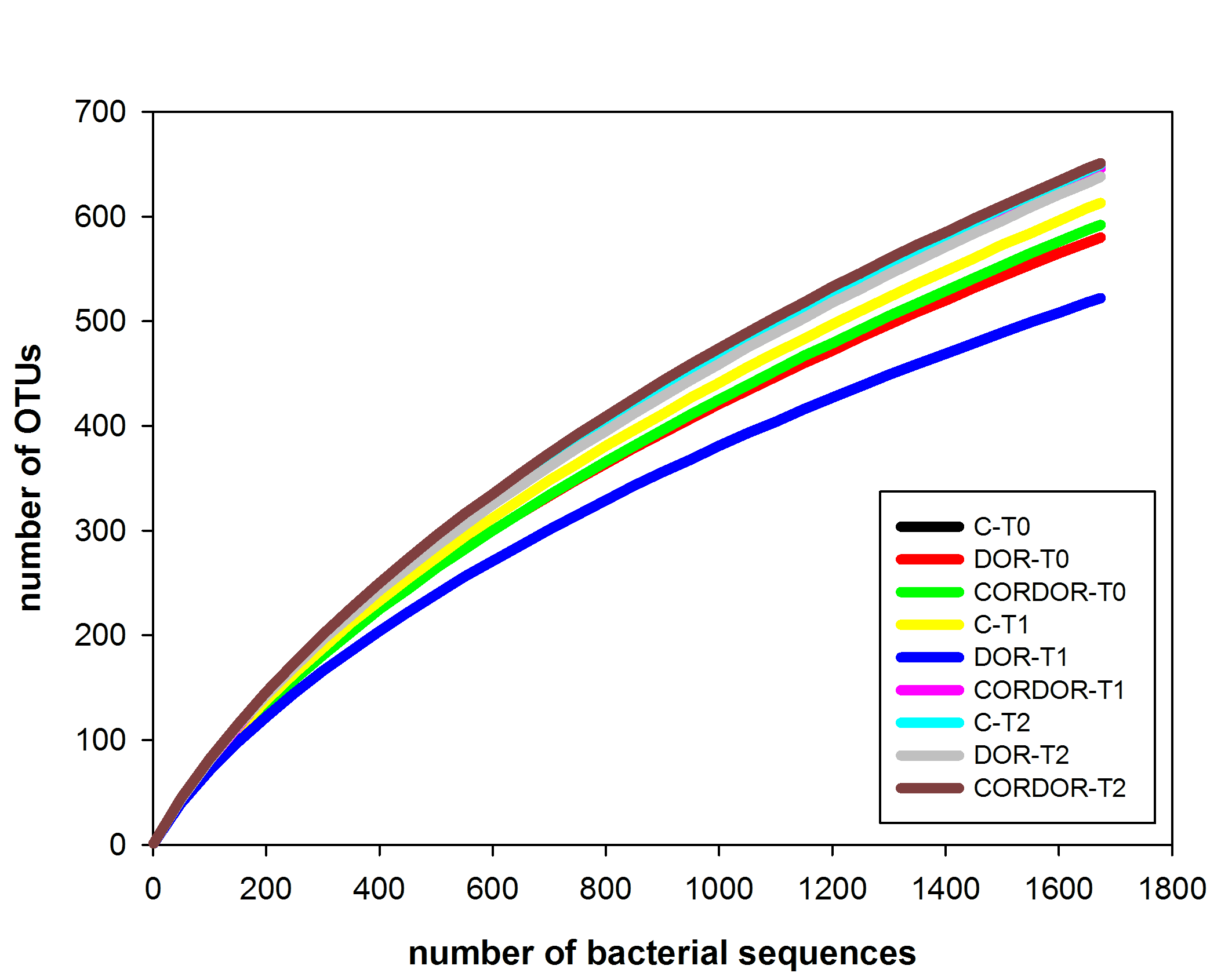

Supplement: Figure S1 — Bacterial rarefaction curves. Rarefaction curves for bacteria obtained from unamended soil (C) and soil amended with untransformed DOR (DOR) or C. floccosa–transformed DOR (CORDOR) at 0 (T0), 30 (T1) and 60 (T2) days. (TIF) [file pone.0103035.s001.tif]

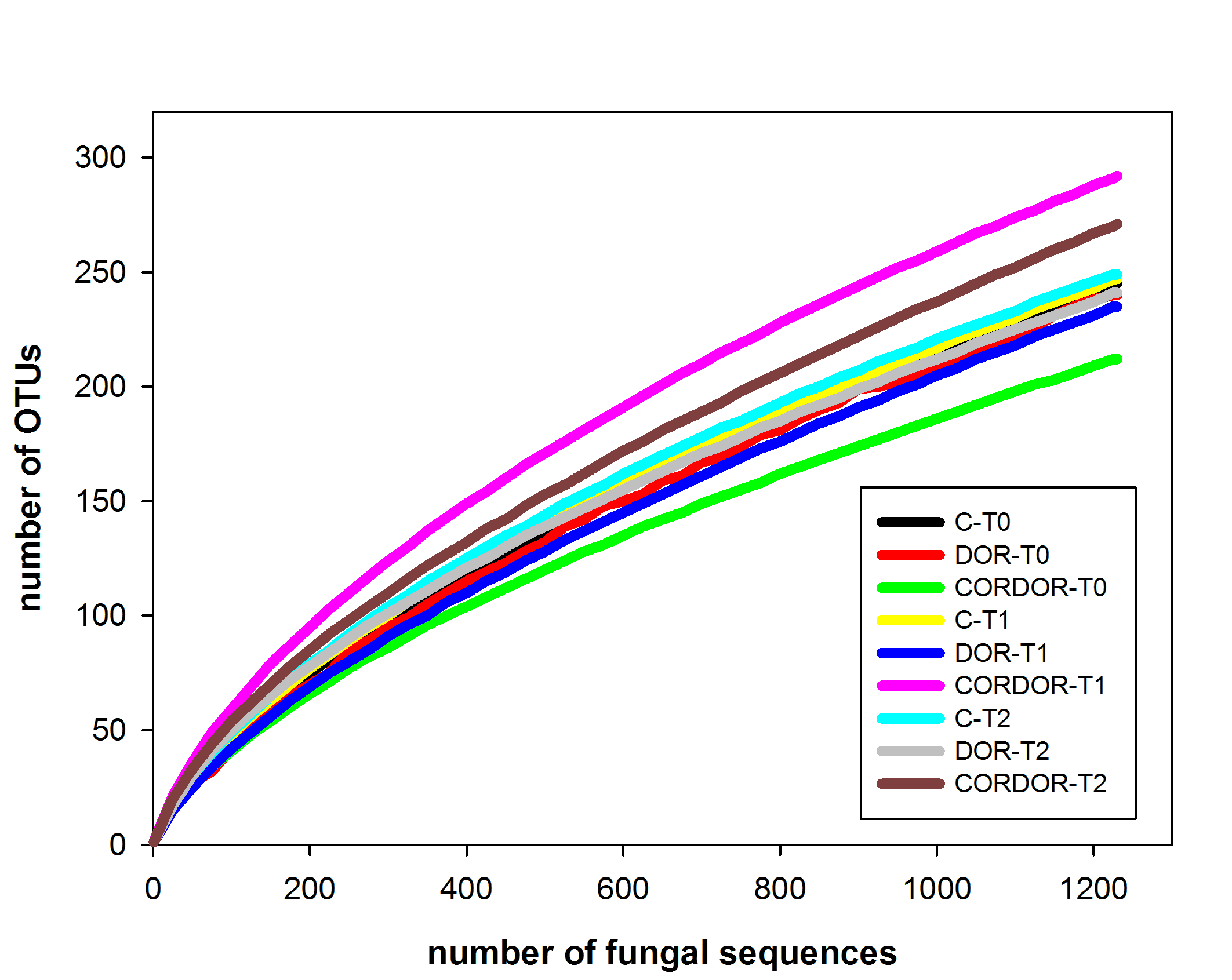

Supplement: Figure S2 — Fungal rarefaction curves. Rarefaction curves for fungi obtained from unamended soil (C) and soil amended with untransformed DOR (DOR) or C. floccosa–transformed DOR (CORDOR) at 0 (T0), 30 (T1) and 60 (T2) days. (TIF) [file pone.0103035.s002.tif]

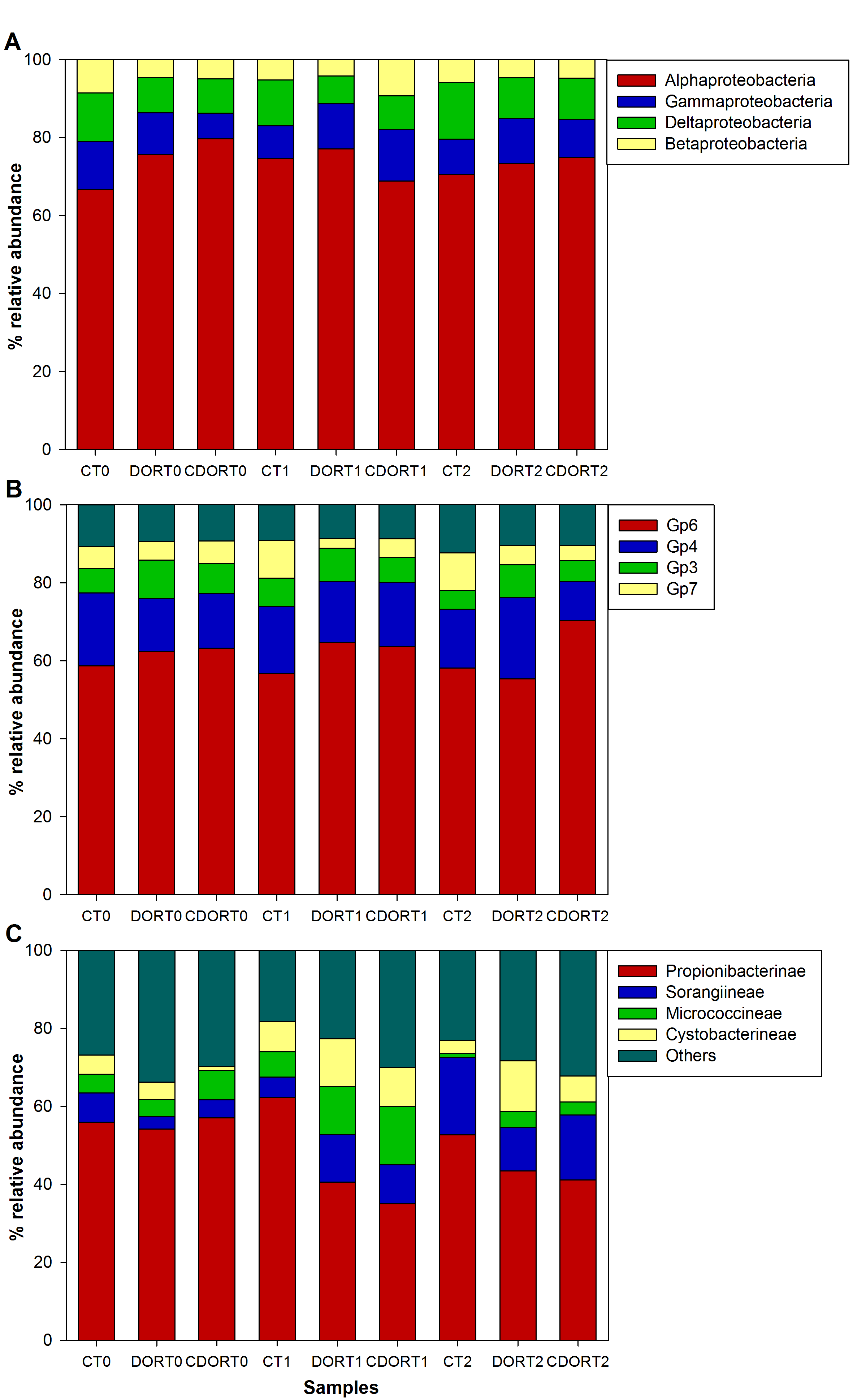

Supplement: Figure S3 — Changes in bacterial community mediated by amendments. Relative abundance of the different Proteobacteria classes (A), Acidobacteria classes (B) and Actinobacteria suborders (C) found in unamended soil (C) and soil amended with untransformed DOR (DOR) or C. floccosa–transformed DOR (CDOR) at 0 (T0), 30 (T1) and 60 (T2) days. (TIF) [file pone.0103035.s003.tif]

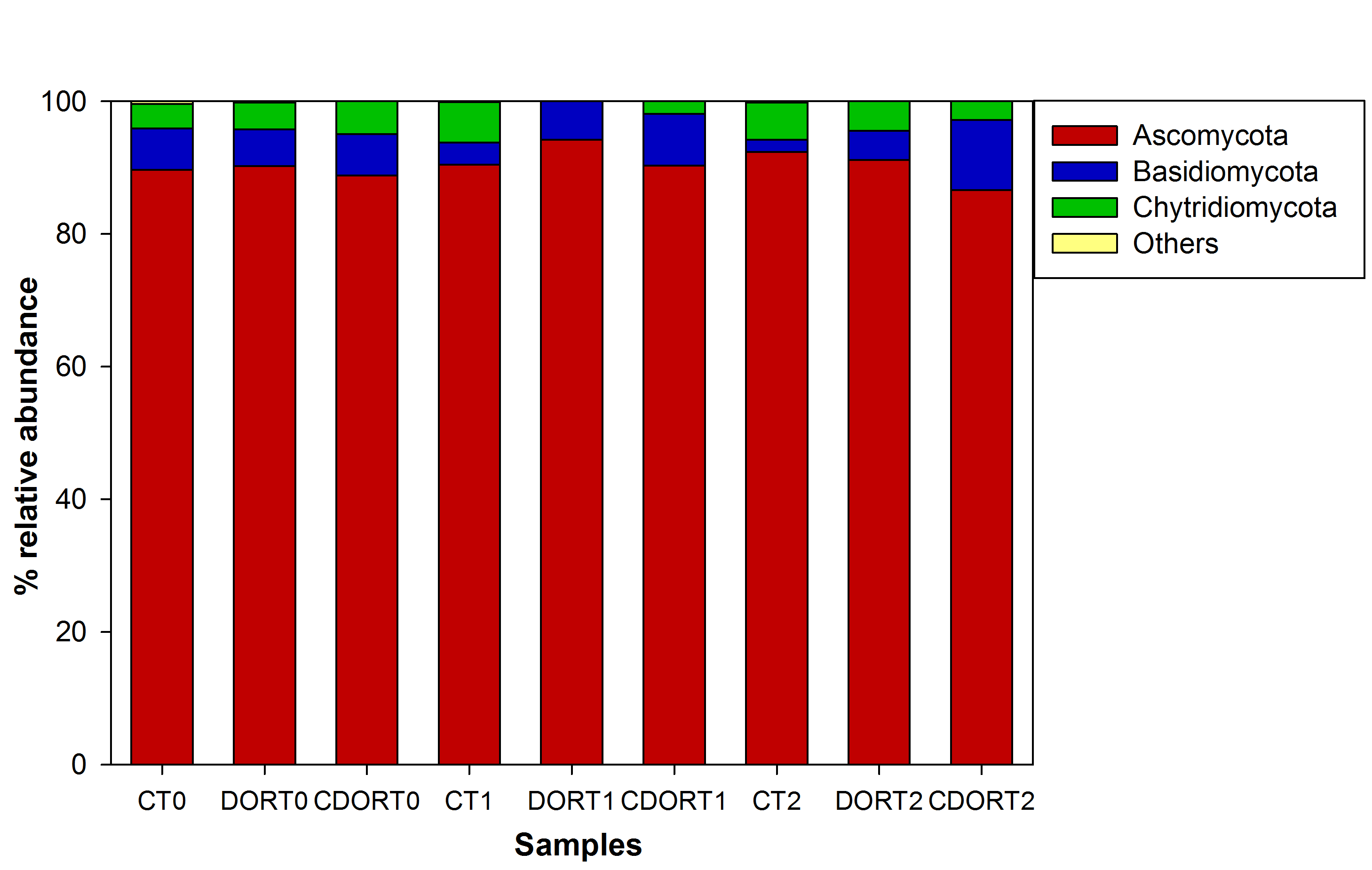

Supplement: Figure S4 — Changes in fungal community mediated by amendments. Relative abundance of the different fungal phyla found in unamended soil (C) and soil amended with untransformed DOR (DOR) or C. floccosa–transformed DOR (CDOR) at 0 (T0), 30 (T1) and 60 (T2) days. (TIF) [file pone.0103035.s004.tif]
