# Supplementary material for: Microbial Diversity of a Mediterranean Soil and Its Changes after Biotransformed Dry Olive Residue Amendment
Source: PLoS One. 2014 Jul 24;9(7):e103035. doi: 10.1371/journal.pone.0103035 (PMC4109964; doi:10.1371/journal.pone.0103035)
Supplement: Table S1 — Soil chemical properties. The chemical properties of the soil used in the study (mean±standard deviation). (DOCX) [file pone.0103035.s005.docx]

**Table S1. Soil chemical properties.** The chemical properties of the soil used in the study (mean±standard deviation).

| **Soil properties** | **Values** |
| --- | --- |
| Clay (%) | 17.15±0.22 |
| Sand (%) | 34.35±0.31 |
| Silt (%) | 48.50±0.52 |
| pH | 8.40±0.01 |
| Total organic carbon (g kg^-1^) | 10.67±0.52 |
| Water soluble carbon (g kg^-1^) | 4.83±0.02 |
| Total nitrogen (g kg^-1^) | 1.52±0.21 |
| P (mg kg^-1^) | 589.78±25.32 |
| K (g kg^-1^) | 8.63±0.21 |
| Ca (g kg^-1^) | 61.90±3.65 |
| Cd (mg kg^-1^) | 1.44±0.08 |
| Cr (mg kg^-1^) | 39.27±0.11 |
| Fe (g kg^-1^) | 20.97±0.13 |
| Cu (mg kg^-1^) | 30.28±0.15 |
| Mg (g kg^-1^) | 17.66±0.11 |
| Mn (mg kg^-1^) | 435.92±19.36 |
| Na (g kg^-1^) | 1.78±0.05 |
| Ni (mg kg^-1^) | 26.88±1.36 |
| Zn (mg kg^-1^) | 73.24±4.32 |
| Pb (mg kg^-1^) | 26.49±1.12 |
| Phenols (g kg^-1^) | 2.16±0.22 |
