# Supplementary material for: Microbial Diversity of a Mediterranean Soil and Its Changes after Biotransformed Dry Olive Residue Amendment
Source: PLoS One. 2014 Jul 24;9(7):e103035. doi: 10.1371/journal.pone.0103035 (PMC4109964; doi:10.1371/journal.pone.0103035)
Supplement: Table S2 — Correlation of carbon sources with principal coordinates. Correlation of carbon sources with the first (PC1) and second principal coordinates (PC2) after principal coordinate analysis (PCoA) of community level physiological profiles (CLPP) from unamended soil and soil amended with untransformed DOR or C. floccosa–transformed DOR at 0, 30 and 60 days. (DOCX) [file pone.0103035.s006.docx]

**Table S2. Correlation of carbon sources with principal coordinates**. Correlation of carbon sources with the first (PC1) and second principal coordinates (PC2) after principal coordinate analysis (PCoA) of community level physiological profiles (CLPP) from unamended soil and soil amended with untransformed DOR or *C. floccosa*–transformed DOR at 0, 30 and 60 days.

| **Substrate name** | **Substrate type** | **PC 1** | **PC 2** |
| --- | --- | --- | --- |
| Water | Control | 0,000 | 0,000 |
| Pyruvic acid methyl ester | Miscellaneous | 0,104 | 0,058 |
| Tween 40 | Polymer | -0,042 | 0,164 |
| Tween 80 | Polymer | 0,186 | 0,111 |
| Alpha-cyclodextrin | Polymer | -0,171 | -0,283 |
| Glycogen | Polymer | -0,174 | -0,157 |
| D-cellobiose | Carbohydrate | -0,075 | -0,372 |
| Alpha-D-lactose | Carbohydrate | -0,186 | -0,108 |
| Beta-methyl-D-glucoside | Carbohydrate | -0,312 | 0,508 |
| D-xylose | Carbohydrate | 0,188 | -0,323 |
| i-erythritol | Carbohydrate | 0,215 | -0,068 |
| D-mannitol | Carbohydrate | -0,112 | -0,041 |
| N-acetyl-D-glucosamine | Carbohydrate | -0,118 | -0,144 |
| D-glucosaminic acid | Carboxylic acid | -0,203 | 0,084 |
| Glucose-1-phosphate | Miscellaneous | -0,062 | 0,081 |
| D,L-alpha-glycerol phosphate | Miscellaneous | 0,027 | -0,081 |
| D-galactonic acid-gamma-lactone | Carboxylic acid | 0,047 | -0,032 |
| D-Galacturonic Acid | Carboxylic acid | 0,598 | 0,199 |
| 2-Hydroxy benzoic acid | Carboxylic acid | -0,007 | -0,008 |
| 4-Hydroxy benzoic acid | Carboxylic acid | 0,021 | 0,031 |
| Gamma-hydroxybutyric acid | Carboxylic acid | -0,001 | -0,003 |
| Itaconic acid | Carboxylic acid | 0,089 | 0,226 |
| Alpha-ketobutyric acid | Carboxylic acid | -0,064 | -0,034 |
| D-malic acid | Carboxylic acid | 0,075 | 0,272 |
| L-arginine | Amino acid | 0,038 | -0,239 |
| L-asparagine | Amino acid | -0,026 | 0,153 |
| L-phenylalanine | Amino acid | -0,148 | -0,111 |
| L-serine | Amino acid | -0,230 | 0,122 |
| L-threonine | Amino acid | -0,064 | 0,040 |
| Glycyl-L-glutamic acid | Amino acid | -0,100 | 0,084 |
| Phenylethylamine | Amine/amide | 0,211 | -0,079 |
| Putrescine | Amine/amide | 0,297 | -0,053 |
