# Supplementary material for: Microbial Diversity of a Mediterranean Soil and Its Changes after Biotransformed Dry Olive Residue Amendment
Source: PLoS One. 2014 Jul 24;9(7):e103035. doi: 10.1371/journal.pone.0103035 (PMC4109964; doi:10.1371/journal.pone.0103035)
Supplement: Table S3 — Identification and abundance of the major bacterial OTUs. Basic information of the bacterial 14 most abundant OTUs and their relative abundance (percent) in unamended soil (C) and soil amended with untransformed DOR (DOR) or C. floccosa–transformed DOR (CORDOR) at 0 (T0), 30 (T1) and 60 (T2) days. (DOCX) [file pone.0103035.s007.docx]

**Table S3. Identification and abundance of the major bacterial OTUs.** Basic information of the bacterial 14 most abundant OTUs and their relative abundance (percent) in unamended soil (C) and soil amended with untransformed DOR (DOR) or *C. floccosa*–transformed DOR (CORDOR) at 0 (T0), 30 (T1) and 60 (T2) days

| **OTU information** | | | | **Soil sample** | | | | | | | | |
| --- | --- | --- | --- | --- | --- | --- | --- | --- | --- | --- | --- | --- |
| **OTU no.** | **Closest relative Eztaxon match**  **(GenBank accession no.), % similarity** | **Class** | **Nº seqs.^1^** | **CT0** | **DORT0** | **CORDORT0** | **CT1** | **DORT1** | **CORDORT1** | **CT2** | **DORT2** | **CORDORT2** |
| **1** | *Skermanella stibiiresistens* (HQ315828), 100 | *Alphaproteobacteria* | 619 | 12.4 | 14.4 | 17.4 | 13.7 | 10.5 | 6.8 | 10.8 | 7.1 | 6.8 |
| **2** | *Microvirga aerophila* (GQ421848), 100 | *Alphaproteobacteria* | 426 | 4.0 | 8.7 | 9.9 | 5.6 | 22.3 | 14.8 | 7.7 | 11.0 | 16.0 |
| **3** | Uncultured *Acidobacteria* (FJ152681), 100 | *Acidobacteria* | 348 | 10.3 | 9.2 | 8.3 | 12.1 | 13.8 | 17.8 | 10.3 | 8.6 | 9.5 |
| **4** | Uncultured *Dongia* sp. (FJ479524), 100 | *Alphaproteobacteria* | 312 | 10.3 | 11.2 | 11.5 | 11.2 | 13.5 | 7.1 | 12.8 | 14.7 | 7.7 |
| **5** | *Nocardioides mesophilus* (EF466117), 100 | *Actinobacteria* | 236 | 20.1 | 14.1 | 19.0 | 14.8 | 5.3 | 4.7 | 15.5 | 3.4 | 3.1 |
| **6** | Uncultured *Phenylobacterium* (AJ292592), 99 | *Alphaproteobacteria* | 186 | 0.0 | 3.8 | 4.3 | 2.2 | 21.0 | 22.0 | 2.7 | 21.5 | 22.6 |
| **7** | *Rhizobium rosettiformans* (EU781656), 100 | *Alphaproteobacteria* | 173 | 2.3 | 2.9 | 0.6 | 1.7 | 53.2 | 15.0 | 1.7 | 18.5 | 4.0 |
| **8** | Uncultured *Acidobacteria* (HM438150), 100 | *Acidobacteria* | 145 | 7.6 | 8.3 | 13.1 | 14.5 | 4.1 | 18.6 | 15.2 | 9.0 | 9.7 |
| **9** | *Skermanella aerolata* (DQ672568), 99 | *Alphaproteobacteria* | 145 | 12.4 | 16.6 | 18.6 | 14.5 | 12.4 | 7.6 | 8.3 | 4.1 | 5.5 |
| **10** | Uncultured *Acidobacteria* (FJ152840), 100 | *Acidobacteria* | 141 | 12.1 | 12.1 | 7.8 | 19.1 | 3.5 | 8.5 | 14.9 | 12.1 | 9.9 |
| **11** | Uncultured *Polyangiaceae* (EU134489), 86 | *Deltaproteobacteria* | 141 | 17.7 | 17.7 | 20.6 | 18.4 | 9.2 | 2.8 | 7.8 | 3.5 | 2.1 |
| **12** | Uncultured *Acidobacteria* (EF688341), 100 | *Acidobacteria* | 138 | 17.4 | 9.4 | 10.1 | 23.9 | 3.6 | 8.0 | 14.5 | 2.2 | 10.9 |
| **13** | Uncultured *Gemmatimonadaceae*(EU881161), 98 | *Gemmatimonadetes* | 138 | 14.5 | 6.5 | 8.0 | 14.5 | 3.6 | 7.2 | 20.3 | 9.4 | 15.9 |
| **14** | Uncultured *Alysiosphaera* (EU133443), 95 | *Alphaproteobacteria* | 134 | 9.7 | 9.0 | 20.9 | 9.7 | 6.7 | 5.2 | 11.9 | 16.4 | 10.4 |

^1^ total number of sequences in normalized samples
