# Supplementary material for: Microbial Diversity of a Mediterranean Soil and Its Changes after Biotransformed Dry Olive Residue Amendment
Source: PLoS One. 2014 Jul 24;9(7):e103035. doi: 10.1371/journal.pone.0103035 (PMC4109964; doi:10.1371/journal.pone.0103035)
Supplement: Table S4 — Identification and abundance of the major fungal OTUs. Basic information of the fungal 14 most abundant OTUs and their relative abundance (percent) in unamended soil (C) and soil amended with untransformed DOR (DOR) or C. floccosa–transformed DOR (CORDOR) at 0 (T0), 30 (T1) and 60 (T2) days. (DOCX) [file pone.0103035.s008.docx]

**Table S4. Identification and abundance of the major fungal OTUs**. Basic information of the fungal 14 most abundant OTUs and their relative abundance (percent) in unamended soil (C) and soil amended with untransformed DOR (DOR) or *C. floccosa*–transformed DOR (CORDOR) at 0 (T0), 30 (T1) and 60 (T2) days

| **OTU information** | | | | **Soil sample** | | | | | | | | |
| --- | --- | --- | --- | --- | --- | --- | --- | --- | --- | --- | --- | --- |
| **OTU**  **no.** | **Closest relative CBS match**  **(GenBank accession no.), % similarity** | **Order** | **Nº seqs^1^** | **CT0** | **DORT0** | **CORDORT0** | **CT1** | **DORT1** | **CORDORT1** | **CT2** | **DORT2** | **CORDORT2** |
| **1** | *Fusarium* sp. (JF740925), 100 | *Hypocreales* | 1547 | 8.0 | 10.5 | 8.7 | 6.5 | 29.4 | 3.3 | 12.6 | 19.1 | 1.9 |
| **2** | *Chaetomium* sp. (JN709486), 100 | *Sordariales* | 1256 | 17.3 | 20.8 | 17.1 | 15.7 | 3.3 | 3.1 | 16.5 | 4.1 | 2.1 |
| **3** | *Preussia terricola* (GQ203725), 99 | *Pleosporales* | 353 | 12.2 | 13.0 | 15.3 | 19.0 | 3.4 | 4.8 | 16.4 | 3.4 | 12.5 |
| **4** | *Stachybotrys chartarum*(AF081468), 100 | *Hypocreales* | 272 | 9.5 | 15.8 | 18.0 | 15.8 | 8.5 | 10.7 | 9.9 | 10.7 | 1.1 |
| **5** | *Rhizopus oryzae* (KC354517), 100 | *Mucorales* | 248 | 29.0 | 2.4 | 53.2 | 10.1 | 0.0 | 3.2 | 0.0 | 1.6 | 0.5 |
| **6** | Uncultured soil fungus (JQ311284), 92 | Incertae sedis | 244 | 18.0 | 23.0 | 16.8 | 12.7 | 5.7 | 4.9 | 11.9 | 2.9 | 4.1 |
| **7** | *Chytridiomycete* (EU873019), 94 | Incertae sedis | 240 | 6.3 | 8.3 | 9.6 | 53.3 | 0.8 | 2.1 | 18.8 | 0.0 | 0.8 |
| **8** | *Aspergillus terreus* (KF278468), 99 | *Eurotiales* | 219 | 21.0 | 19.2 | 21.3 | 15.6 | 2.7 | 2.7 | 14.7 | 1.4 | 1.4 |
| **9** | *Cryptococcus* sp. (DQ531950), 99 | *Tremellales* | 184 | 0.0 | 1.1 | 0.0 | 0.0 | 22.2 | 43.5 | 0.0 | 8.7 | 24.5 |
| **10** | Uncultured fungus (KC558360), 100 | Incertae sedis | 169 | 1.2 | 0.6 | 0.0 | 26.0 | 8.9 | 32.5 | 8.9 | 4.1 | 17.8 |
| **11** | Uncultured *Podospora* sp. (GU055536), 100 | *Sordariales* | 159 | 1.3 | 0.0 | 0.6 | 0.0 | 0.0 | 18.9 | 1.9 | 18.2 | 59.1 |
| **12** | *Cercophora sordarioides* (AY780064), 99 | *Sordariales* | 157 | 0.0 | 0.0 | 7.0 | 0.6 | 1.3 | 38.2 | 0.0 | 9.6 | 43.3 |
| **13** | *Coprotus ochraceus* (KC012673), 99 | *Thelebolales* | 154 | 0.0 | 0.0 | 0.0 | 0.0 | 1.3 | 5.2 | 0.0 | 40.9 | 52.6 |
| **14** | *Ascobolus* sp. (AY500527), 98 | *Pezizales* | 149 | 22.8 | 16.8 | 8.7 | 4.0 | 1.3 | 16.1 | 12.8 | 2.7 | 14.8 |

^1^ total number of sequences in normalized samples
